# SynthSR: A public AI tool to turn heterogeneous clinical brain scans into high-resolution T1-weighted images for 3D morphometry

**DOI:** 10.1126/sciadv.add3607

**Published:** 2023-02-01

**Authors:** Juan E. Iglesias, Benjamin Billot, Yaël Balbastre, Colin Magdamo, Steven E. Arnold, Sudeshna Das, Brian L. Edlow, Daniel C. Alexander, Polina Golland, Bruce Fischl

**Affiliations:** ^1^ Athinoula A. Martinos Center for Biomedical Imaging, Massachusetts General Hospital and Harvard Medical School, Boston, MA, USA.; ^2^ Centre for Medical Image Computing, Department of Computer Science, University College London, London, UK.; ^3^ Computer Science and Artificial Intelligence Laboratory (CSAIL), Massachusetts Institute of Technology, Cambridge, MA, USA.; ^4^ Department of Neurology, Massachusetts General Hospital and Harvard Medical School, Boston, MA, USA.; ^5^ Center for Neurotechnology and Neurorecovery, Massachusetts General Hospital, Boston, MA, USA.

## Abstract

Every year, millions of brain magnetic resonance imaging (MRI) scans are acquired in hospitals across the world. These have the potential to revolutionize our understanding of many neurological diseases, but their morphometric analysis has not yet been possible due to their anisotropic resolution. We present an artificial intelligence technique, “SynthSR,” that takes clinical brain MRI scans with any MR contrast (T1, T2, etc.), orientation (axial/coronal/sagittal), and resolution and turns them into high-resolution T1 scans that are usable by virtually all existing human neuroimaging tools. We present results on segmentation, registration, and atlasing of >10,000 scans of controls and patients with brain tumors, strokes, and Alzheimer’s disease. SynthSR yields morphometric results that are very highly correlated with what one would have obtained with high-resolution T1 scans. SynthSR allows sample sizes that have the potential to overcome the power limitations of prospective research studies and shed new light on the healthy and diseased human brain.

## Teaser

A public AI tool turns clinical brain MRI of any resolution/contrast into 1mm T1 scans compatible with neuroimaging pipelines.

## INTRODUCTION

Neuroimaging with MRI is one of the most useful tools available to study the human brain in vivo. Open-source neuroimaging software packages like FreeSurfer (*1*), FSL (*2*), SPM (*3*), and AFNI (*4*) have enabled researchers around the world to conduct brain studies in an automated fashion, e.g., to characterize brain structure and function in healthy aging and in diseases like Alzheimer’s. These tools have also increased reproducibility of results, particularly when combined with publicly available datasets such as Alzheimer’s Disease Neuroimaging Initiative (ADNI) (*5*), the Human Connectome Project (*6*), or the UK Biobank (*7*).

The automated processing methods in current neuroimaging tools require magnetic resonance imaging (MRI) scans acquired with high, isotropic resolution (typically 1 mm) to minimize errors in three-dimensional (3D) analyses such as segmentation (*8*–*10*) or registration (*11*–*16*). In addition, several methods have requirements in terms of MR contrast. For example, FreeSurfer requires T1-weighted scans. For this reason, most modern research neuroimaging studies include a structural MRI acquisition that fulfills such resolution and MR contrast requirements—often a 1-mm isotropic, T1-weighted scan acquired with the ubiquitous 3D magnetization prepared - rapid gradient echo (MPRAGE) pulse sequence (*17*).

However, the vast majority of MRI scans in the world are acquired for clinical purposes and do not satisfy the aforementioned criteria. In the clinic, physicians typically prefer 2D acquisitions that yield fewer slices (e.g., 20 to 30) with large spacing (5 to 7 mm) and high in-plane resolution (under 1 mm), often acquired with the widespread turbo spin echo (TSE) sequence (*18*). This type of acquisition reduces the time that is required to inspect the images and is less sensitive to motion artifacts, which is crucial for patients whose neurological diseases make it challenging for them to lie still in the MRI scanner.

The inability to compute morphometric measurements from clinical scans for use in neuroimaging research is a major barrier to progress in this field, as it precludes the analysis of millions of scans that are currently sitting in picture archiving and communication systems (PACS) in hospitals worldwide. For example, approximately 10 million brain MR exams were performed in the United States in 2019 alone (*19*). These figures are huge compared with even the largest neuroimaging MRI studies and meta-studies (*20*), and could yield sample sizes with the potential to revolutionize our understanding of neurological conditions and genetic linkages, compared with typically underpowered prospective research studies (*21*).

Artificial intelligence (AI) techniques—particularly emerging deep machine learning (ML) models for image synthesis and super-resolution (SR)—have the potential to bridge the gap between clinical and research-grade brain MRI scans. Given a scan of a certain (non-T1) MR contrast and low (non-isotropic) resolution, these techniques can be used to generate a new imaging volume that has T1-like contrast (via synthesis) and high, isotropic resolution (via SR).

There are many existing techniques for synthesis and SR of MRI, based both on classical and deep ML methods. Modern SR methods are almost exclusively based on deep learning (*22*, *23*), specifically convolutional neural networks (CNNs). These typically capitalize on large amounts of paired low- and high-resolution images (LR/HR) to learn mappings from the former to the latter (*24*–*26*). Further refinement of the output can be achieved by enhancing the architecture with adversarial losses (*27*), i.e., by trying to fool a discriminator that is trained to separate real HR images from enhanced LR images (*28*–*30*). Paired data are easy to obtain, by taking HR images and downsampling them to obtain LR counterparts.

Similar to SR, modern synthesis methods are based on CNNs seeking to learn a mapping between a source and a target modality—often enhanced with adversaries, too, trained to discriminate real and synthetic images. While obtaining perfectly paired data is more difficult than in SR, this limitation has been mitigated with unpaired techniques based on generative adversarial networks (GANs), which seek to generate synthetic images of the target modality that are difficult to discriminate from real ones by an auxiliary CNN (*27*). A representative example is the CycleGAN framework (*31*), which combines GANs with cycle consistency, i.e., mapping an image to a target domain and then back to the source should be close to the identity operator. However, GANs are typically used in combination with supervised losses based on voxel-wise errors, since they underperform when used in isolation (*32*). Driven by applications like estimation of missing modalities and synthesis of computed tomography (CT) from MRI for attenuation correction in positron emission tomography (PET), many methods have been developed for image synthesis in brain MRI (*33*–*39*).

Unfortunately, three main roadblocks have precluded the application of deep learning SR and synthesis techniques to clinical brain MRI data. First, the domain shift: The performance of CNNs quickly decreases when the resolution or MR contrast of the input diverges from the data that the network was trained on (*40*). This gap is particularly problematic in clinical brain MRI, due to the huge diversity in orientation, resolution, and MR contrast of acquisitions, both within and across centers. Data augmentation (*41*) and domain adaptation techniques (*42*) mitigate the problem but have not been able to close the gap. Self-supervised SR techniques exploit the high-frequency content within slices to learn to super-resolve the LR in orthogonal views across slices (*43*), but cannot tackle the synthesis problem, since they only have access to the target modality. The second obstacle is the need to retrain. Even if training data were available for every possible combination of orientation, contrast, and resolution, different MRI exams with different sets of pulse sequences would require additional runs of training or, at least, domain adaptation. Self-supervised methods also suffer from this limitation. Therefore, there are currently no methods that can be used “out of the box” to process heterogeneous datasets. The third barrier is the modeling of pathology: To the best of our knowledge, no existing SR or synthesis method is robust to the wide variation of pathology that one encounters in a PACS.

Here, we present our neural network SynthSR, which we distribute with FreeSurfer. SynthSR turns a clinical brain MRI scan of any orientation, resolution and contrast into a 1-mm isotropic 3D MPRAGE. This synthetic MPRAGE can be subsequently analyzed with any existing tool for 3D image analysis of brain MRI, e.g., registration or segmentation. SynthSR is an evolution of our previous tool (*44*), which could be trained to process images of predefined resolution and contrast using synthetic data, yet suffered from the three limitations described above. Our new tool, on the other hand, (i) combines a domain randomization (DR) approach (*45*) with a generative model of brain MRI to support scans of any resolution and contrast out of the box without retraining, (ii) produces more realistic images via an auxiliary segmentation task, and (iii) handles abnormalities by inpainting them with normal-looking tissue. Because most standard neuroimaging tools like FreeSurfer or FSL cannot cope with pathology (particularly large lesions like some tumors or strokes), inpainting them with healthy tissue enables direct subsequent analysis with these tools. This approach is common, e.g., in the multiple sclerosis literature, where white matter lesions are filled with white matter–like intensities before 3D morphometry with packages like FreeSurfer or SPM (*46*).

SynthSR is publicly available and can be easily used by downloading FreeSurfer (https://surfer.nmr.mgh.harvard.edu/fswiki/DownloadAndInstall) and executing the command

mri_synthsr --i [clinical_scan] --o [synthetic_1mm_isotropic_t1]which only takes a few seconds to run on a graphics processing unit (GPU) or approximately 15 s on a modern desktop computer without a GPU.

## RESULTS

### Image segmentation and volumetry with existing tools

One of the main use cases of SynthSR is automated volumetry of regions of interest (ROIs) from MRI scans that do not satisfy the requirements of the packages that are often used for this purpose. We used SynthSR to process the 9146 brain MRI scans in the Massachusetts General Hospital (MGH) dataset, which includes nearly uncurated data from 1110 patients with neurology visits at MGH; we note that we left out scans with more than three dimensions (e.g., diffusion-weighted MRI) or with intracranial volume (ICV) under 1.1 liters (typically with partial field of view); further details can be found in Materials and Methods.

**Fig. 1.**
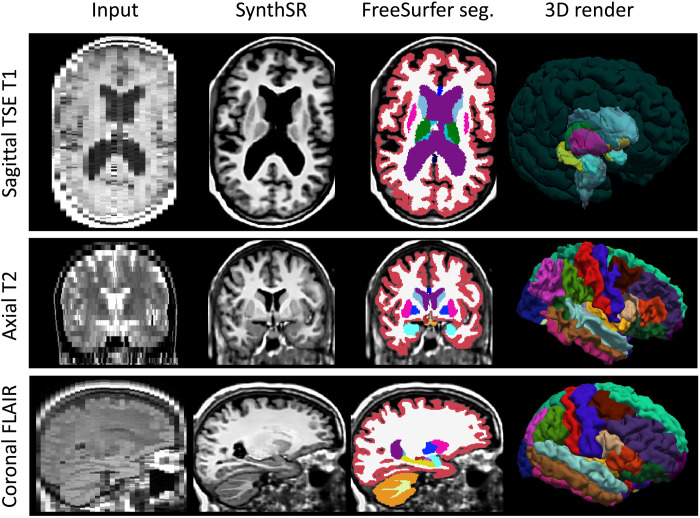
Examples of inputs and outputs of SynthSR from the MGH dataset. Each of the inputs has been acquired with a different orientation (axial, sagittal, and coronal), slice spacing (6, 5, and 4 mm), and MR contrast (turbo spin echo T1, T2, FLAIR); we visualize them in orthogonal view to illustrate their low-resolution out of plane. SynthSR produces a synthetic 1-mm isotropic MPRAGE in all cases, which is compatible with nearly every existing brain MR image analysis method. The third column shows the automated segmentation of the synthetic MPRAGE obtained with FreeSurfer, and the last column shows three-dimensional (3D) renderings of (top to bottom) the subcortical segmentation, parcellated white matter surface, and parcellated pial surface. We emphasize that all images were processed with the same neural network using SynthSR out of the box, without retraining.

Examples of the synthetic outputs are shown in Fig. 1. We segmented these synthetic images with FreeSurfer and compared the volumes derived from these segmentations with ground-truth estimates for a subset of 435 scans from 41 patients who also had a 1-mm isotropic T1-weighted scan. Correlations at the scan and subject level (computed as the median of the volumes across available scans within a session) are shown in Table 1. The correlations at the single scan level are strong (~0.8) for tissue classes (white matter, cortical gray matter, and subcortical gray matter), nearly perfect for the ventricles, and moderate to strong for individual subcortical ROIs—ranging from 0.47 for the pallidum (which is very difficult to segment in T1 due to the lack of contrast) to 0.76 for the hippocampus. When the measurements from a single session are aggregated into a single estimate, these correlations increase considerably, becoming very strong (~0.9) for nearly every tissue class and subcortical ROI, except for the putamen and pallidum—for the aforementioned reasons.

**Table 1.** Correlation with ground-truth volumes. Pearson correlations between volumes obtained with FreeSurfer from 1-mm isotropic T1s and from the synthetic MPRAGEs computed with SynthSR from clinical acquisitions of heterogeneous orientation, resolution, and contrast. The correlations at the scan level are computed with and without the auxiliary segmentation loss to analyze the impact of this component of our method. The correlations at the subject level use volume estimates computed as the median across available scans for each subject (using the full SynthSR model, i.e., with the segmentation loss). All correlations are strongly significant (*P* < 10^−7^ when using the full model).

Region level	White matter	Cortical gray matter	Subcortical gray matter	Ventricles	Hippocampus	Amygdala	Thalamus	Putamen	Pallidum
Scan level (*n* = 435)	0.79	0.83	0.77	0.99	0.76	0.60	0.72	0.60	0.47
Scan level (ablated segmentation task)	0.79	0.79	0.76	0.99	0.74	0.54	0.69	0.56	0.40
Subject level (*n* = 41)	0.91	0.93	0.91	0.99	0.89	0.90	0.90	0.75	0.72

To study the contribution of the auxiliary segmentation task to the performance SynthSR, we performed an ablation study where the auxiliary segmentation task was disregarded during training. When this alternative model was used, correlations decreased for almost all ROIs, especially those with faint or convoluted boundaries (e.g., cortex, amygdala, and pallidum). This result highlights the importance of the segmentation loss for accurately synthesizing such brain regions.

To study the performance of SynthSR as a function of resolution, we stratified the correlations by resolution using groups of scans with similar slice spacing. The results are shown in Table 2 and show a clear pattern of decrease in correlation with growing spacing. Some larger ROIs like the ventricles or cortical gray matter are more robust to slice spacing than smaller ROIs like the amygdala or pallidum. We also note that aggregating results at the subject level (i.e., bottom row of Table 1) yields, on average, higher correlations than the scans with smaller spacing on their own (top row of Table 2).

**Table 2.** Performance as a function of slice spacing. Pearson correlations between volumes obtained with FreeSurfer from 1-mm isotropic T1s and from the synthetic MPRAGEs were computed with SynthSR from clinical acquisitions of heterogeneous orientation and contrast as a function of slice spacing. All correlations are strongly significant (*P* < 10^−3^).

Region slice spacing	White matter	Cortical gray matter	Subcortical gray matter	Ventricles	Hippocampus	Amygdala	Thalamus	Putamen	Pallidum
Up to 4 mm (*n* = 197)	0.92	0.84	0.90	0.99	0.89	0.80	0.92	0.73	0.67
Between 4 and 6 mm (*n* = 110)	0.71	0.84	0.71	0.99	0.69	0.55	0.63	0.50	0.41
More than 6 mm (*n* = 128)	0.68	0.80	0.64	0.99	0.56	0.37	0.59	0.49	0.27

**Fig. 2.**
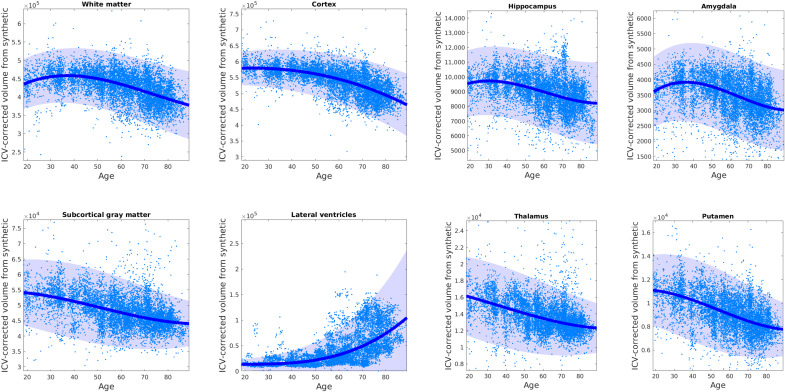
Brain volume aging trajectories derived from clinical data using SynthSR combined with FreeSurfer. Clinical scans were processed with SynthSR and then segmented with FreeSurfer to obtain ROI volumes as well as an estimate of the intracranial volume (ICV). The ROI volumes were corrected by ICV and sex and regressed against age using a Laplace distribution with location and scale modeled with a spline with six knots. The Laplace distribution provides a more robust fit than a Gaussian. The median trajectory is overlaid on the individual (ICV- and gender-corrected) volumes; the 95% confidence interval is shaded. The volumes are computed at the scan level; trajectories computed at the subject level are shown in fig. S1.

We then used the segmentations from the MGH dataset to test whether we could replicate well-established atrophy patterns due to aging. Figure 2 shows individual points (at the scan level) and regressed median trajectories with confidence intervals for different tissue classes and subcortical ROIs. Trajectories at the subject level are shown in fig. S1. The trajectories in Fig. 2 are remarkably similar to those from a recent meta-analysis with more than 100,000 HR isotropic scans (*47*): They correctly capture the peak of the white matter at about 30 years, the earlier decline of the gray matter, and the highly nonlinear trajectory of the ventricles. Despite using clinical scans with large spacing, our method also produces trajectories for subcortical ROIs that are highly consistent with previously published studies relying on thousands of 1-mm isotropic T1 scans (*48*, *49*), showing, e.g., earlier atrophy of the thalamus compared with hippocampus or amygdala.

### Detecting disease-induced atrophy: Alzheimer’s disease volumetry

The following experiment seeks to illustrate the ability of SynthSR to preserve the effects of disease on the shapes and volumes of ROIs when processing scans with large slice spacing while demonstrating its compatibility with packages other than FreeSurfer. For this purpose, we combined SynthSR and FSL to detect hippocampal atrophy in Alzheimer’s disease (AD). Using this application has two advantages. First, hippocampal atrophy is a well-established biomarker of this type of dementia (*50*–*52*), so we know what results to expect. Second, we can use publicly available scans from the ADNI dataset (*5*), which has the advantage of including 5-mm axial fluid attenuated inversion recovery (FLAIR) scans (which we feed to SynthSR) and 1-mm isotropic MPRAGE scans (which we use as ground truth).

In this experiment, we used a sample with 50 randomly selected AD cases and 50 randomly selected controls (47 males, 53 females; aged 72.9 ± 7.6 years). This sample size is representative of modest-sized neuroimaging research studies and ensures that the analysis is not overpowered to the point that even poor hippocampal segmentations lead to very small *P* values. We used FSL to compute segmentations and derive volumes from the synthetic scans estimated from the FLAIR acquisitions; the volumes estimated from the real MPRAGEs directly with FSL were used as ground truth. We emphasize that hippocampal volumetry with this dataset is a very challenging task: Because the major axis of the hippocampus is approximately parallel to the axial plane, this ROI is visible in very few slices (often just two to four).

**Fig. 3.**
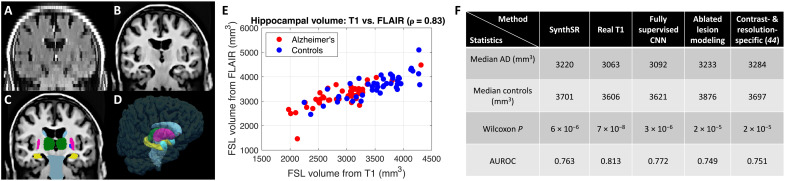
Hippocampal volumetry of AD with SynthSR and FSL. (**A**) Coronal view of sample 5-mm axial FLAIR scan. (**B**) SynthSR. (**C**) Segmentation of (B) with FSL. (**D**) 3D rendering of (C). (**E**) Scatterplot of hippocampal volumes computed with FSL from the 1-mm isotropic T1 scans and from the synthetic MPRAGEs (Pearson correlation: 0.83, *P* < 10^−24^). (**F**) Nonparametric statistics for hippocampal volume of AD versus controls: medians, *P* value for Wilcoxon rank sum test, and AUROC.

Figure 3 (A to D) shows the output of SynthSR and the subsequent FSL segmentation for a sample scan. Qualitatively, SynthSR recovers many of the missing high-frequency details and enables accurate segmentation with FSL. Figure 3E shows a scatterplot for the ground truth and estimated hippocampal volumes (note that we excluded four outliers for which the FSL segmentation failed on the real 1-mm T1 scan). The plot reveals a strong correlation (ρ = 0.83, *P* < 10^−24^) between the volumes estimated from the real T1s and the synthetic MPRAGEs. The latter have a slight positive bias due to the smoothing that is introduced by SynthSR when interpolating between distant MRI slices.

Because the hippocampal volumes of both AD subjects and controls (as estimated from the real T1s) did not follow Gaussian distributions—*P* < 0.05 for a Shapiro-Wilk test (*53*)—we used nonparametric statistics to compare AD versus controls. In this setting, we compared SynthSR against (i) analysis of the real 1-mm MPRAGE scans, which provides a ceiling for the performance of the synthesis methods and enables quantification of the gap with respect to such ceiling; (ii) a fully supervised U-net, trained with spatially aligned pairs of real scans, which provides a ceiling for the performance of synthesis-based methods; (iii) an ablation of the lesion modeling component of SynthSR, which enables us to assess whether this building block has an impact on the ability of the tool to detect disease-induced atrophy; and (iv) our previous version of the tool (*44*)—plus the segmentation loss (for fair comparison)—which enables us to assess whether DR in SynthSR incurs a decrease in performance with respect to training with simulations of the specific resolution and contrast of the FLAIR scans.

Figure 3F shows the median of the two groups, the *P* value for a nonparametric Wilcoxon rank sum test (*54*), and the area under the receiver operating characteristic curve (AUROC), which quantifies the separation between the two classes in a nonparametric setting. Despite the sparse slices in an orientation almost parallel to the major axis of the hippocampus (as mentioned above, it often appears in only two to four slices), SynthSR yields very strong discriminative power between the two groups, almost as much as the real 1-mm isotropic T1s. Our approach only loses 12% of the separation of the medians (481 versus 543 mm^3^) and five AUROC points (0.76 versus 0.81) and provides a very strong *P* value (~10^−6^ versus ~10^−8^). Compared with the fully supervised U-net, which has access to the real FLAIR intensities in training, SynthSR only loses two AUROC points. Ablating the lesion modeling component of SynthSR slightly worsens the results (AUROC = 0.75); this result supports the hypothesis that the inpainting step (which is crucial in many applications of SynthSR, as shown in the rest of experiments below) does not have a negative impact on the sensitivity of the method to disease-induced atrophy. Last, training on synthetic images that are simulated to resemble the contrast and resolution of the 5-mm axial FLAIR scans works slightly worse than fully randomizing resolution and contrast during training. We hypothesize that this is because DR mitigates the inaccuracies of the model in terms of, e.g., slice selection profiles, noise, or bias field.

### Improving registration of clinical scans: Application to MRI of brain tumors

SynthSR can also improve the accuracy of image registration of clinical brain MRI scans. Image registration (*55*), i.e., spatial alignment of pairs of images, has found wide application in neuroimaging, e.g., in areas like longitudinal analysis (*56*), fusion of multimodality scans (*57*), or creation of population atlases (*58*). One important application of registration is the spatial mapping of neuroanatomical correspondences in preoperative and follow-up scans of patients with glioma, as finding features in the former that can predict tumor infiltration and recurrence is crucial for guiding treatment (*59*). However, this registration can be difficult due to differences in orientation, resolution, MR contrast, and tumor size and appearance of the preoperative and follow-up scans—a problem that SynthSR can mitigate by synthesizing 1-mm isotropic MPRAGE images with inpainted tumors.

We applied SynthSR to Brain Tumor Sequence Registration (BraTS-Reg), a recently released dataset of multimodal brain scans of patients with glioma, acquired pre- and postoperatively, which includes manual annotations of corresponding landmarks (*60*). The dataset includes scans from 140 patients and 1252 landmarks. We note that we detected some outliers in the landmarks and are working with the authors of the article to amend them. The results presented here are for a subset of 1075 landmarks that passed our quality control. We provide the list of landmarks that passed quality control in the Supplementary Materials.

**Fig. 4.**
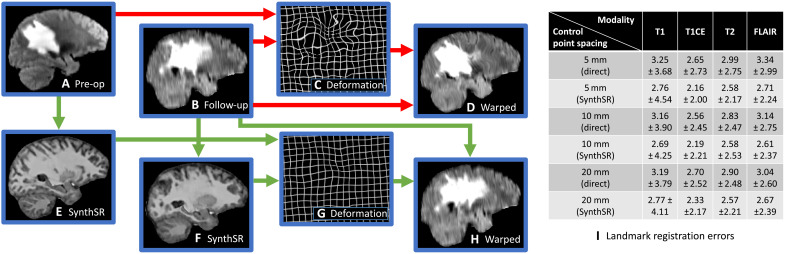
Alignment of preoperative and follow-up brain MRI scans of patients with glioma (BraTS-Reg dataset) using direct registration (red arrows) and SynthSR (green arrows). (**A**) Sagittal slice of the FLAIR scan of a sample subject (axial acquisition). (**B**) Approximately corresponding slice of follow-up scan. (**C**) Deformation field estimated from (A) and (B) with NiftyReg (5-mm control point spacing). (**D**) Warped follow-up scan. (**E** and **F**) SynthSR output of the preoperative and follow-up scans. (**G**) Deformation field estimated from the synthetic scans. (**H**) Warped follow-up scan using the deformation field computed with the synthetic images. (**I**) Mean and SD of landmark error (in millimeters) for different control point spacings and MR contrasts, registering the scans directly versus registering the output of SynthSR. The *P* values for nonparametric Wilcoxon tests comparing the medians are strongly significant in all cases (10^−9^ < *P* < 10^−3^).

The set of modalities is fixed for all subjects and time points: T1, T1 with contrast enhancement (T1CE), T2, and FLAIR; however, the resolution varies across contrasts and time points, as BraTS-Reg is a “multi-institutional dataset consisting of scans acquired under routine clinical conditions, and hence reflecting very heterogeneous acquisition equipment and protocols, affecting the image properties” (*60*). An example of the application of SynthSR to this dataset is shown in Fig. 4 (A and B), where our method successfully upscales the axial FLAIR scans to 1-mm isotropic resolution while synthesizing MPRAGE contrast and inpainting abnormalities.

We then used the well-established neuroimaging registration package NiftyReg to register the preoperative and follow-up scans. NiftyReg implements a robust affine alignment method based on block matching (*61*) as well as a nonlinear diffeomorphic registration model based on stationary velocity fields parameterized by grids of control points (*62*). We registered the scans with the local normalized cross-correlation metric (which is commonly used in MRI due to its robustness to bias fields) and three different control point spacings (5, 10, and 20 mm), which model different levels of freedom of the nonlinear transform, i.e., different compromises between accuracy (lower spacing) and robustness (higher spacing). Default values were used for all other parameters.

When the original scans are registered directly with NiftyReg, the differences in resolution and appearance of the tumor area (Fig. 4, A and B) make registration difficult. NiftyReg erroneously tries to match tumor boundaries and other partial voluming artifacts, thus yielding highly convoluted deformation fields (Fig. 4, C and D). SynthSR mitigates this problem by computing the registration from synthetic images of isotropic resolution and similar appearance (Fig. 4, E and F), which yields much more regular deformation fields (Fig. 4, G and H). This approach reduces the average landmark error after registration for every combination of control point spacings and MR contrasts, between 10 and 20% (Fig. 4C). We note that the SD of the error increased for the T1 scans but decreased for the T1CE, T2, and FLAIR scans. In some cases, SynthSR’s robustness enables us to obtain better results with more aggressive (lower) control point spacings, compared with using the original images (e.g., for T1CE or FLAIR images).

**Fig. 5.**
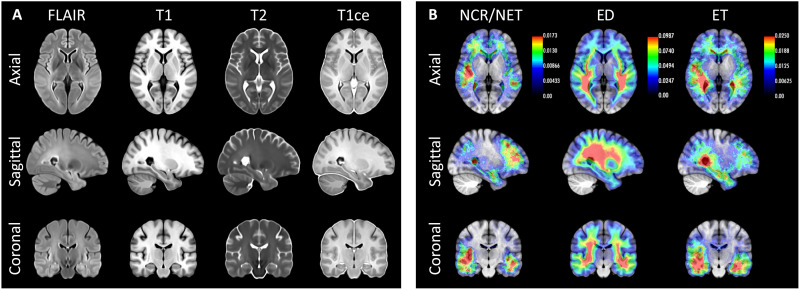
Population templates and probabilistic maps of tumor subregions for the BraTS dataset. (**A**) Axial, sagittal, and coronal slices of the group-wise median templates of the four available modalities. (**B**) Average spatial distribution maps of necrotic and non-enhancing tumor core (NCR/NET), peritumoral edema (ED), and gadolinium-enhancing tumor (ET). The orientation of the images follows radiological convention. The overlaid heatmaps show the probability of observing each tumor class at every voxel, as encoded in the color bars.

We also applied SynthSR to the original BraTS dataset (*63*), which consists of 1251 cases with manually traced low- and high-grade gliomas, and the same four MR contrasts as BraTS-Reg. Using the synthetic MPRAGEs to compute registrations, we estimated an unbiased atlas (*64*) of patients with glioma with the four MR contrasts, as well as probabilistic maps of the manually labeled tumor regions: enhancing tumor, peritumoral edema, and the necrotic and non-enhancing tumor core. Such templates and probability maps are useful in neuroimaging studies for spatial normalization and voxel-wise analysis. The median atlas is shown in Fig. 5. SynthSR overcomes the differences in resolution and the presence of tumors, therefore enabling precise co-registration leading to sharp atlases—particularly in the axial plane, which is the predominant direction of acquisition in the dataset. The accurate alignment also reveals a frontotemporal pattern in the spatial distribution of the gliomas that has been reported in the literature (*65*) and also captures the tendency of gliomas to infiltrate the white matter.

### Segmentation and registration of stroke MRI

Morphometry of brain MRI of patients with stroke is important in the discovery of imaging biomarkers of outcome (*66*) and to study post-stroke dementia, which is becoming increasingly common as stroke mortality rates decrease (*67*). Unfortunately, there are (to the best of our knowledge) no morphometric tools that can readily cope with the abnormal distributions of shape and image intensity distribution caused by stroke, which forces studies to discard many cases that do not pass quality control (*68*)—not only decreasing sample sizes but also introducing a potential selection bias.

**Fig. 6.**
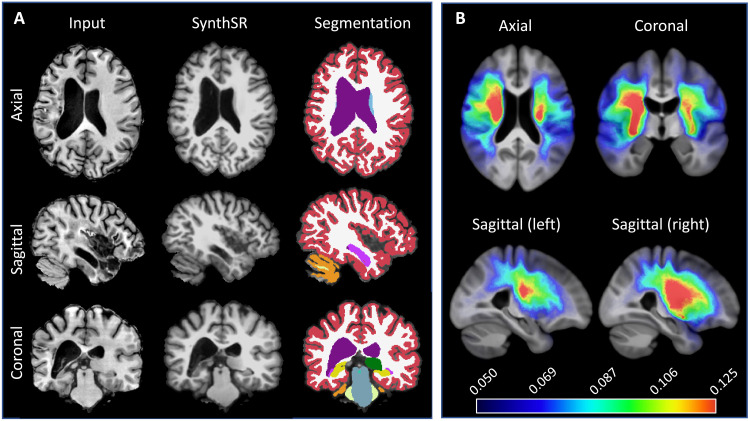
Application of SynthSR to stroke dataset (ATLAS). (**A**) Axial, sagittal, and coronal slices of a sample case, along with the output of SynthSR and its segmentation with FreeSurfer. (**B**) Orthogonal slices of the group-wise median template, along with the average spatial distribution maps of stroke lesions. As in previous figures, the orientation of the images follows radiological convention.

As in the previous experiment, SynthSR can remove differences in resolution, MR contrast, and lesion size and appearance by synthesizing 1-mm MPRAGE scans with inpainted lesions. To evaluate SynthSR in this context, we applied it to ATLAS (*69*, *70*), a publicly available dataset with 655 T1 brain MRI scans of patients with stroke, which includes manually segmented lesion masks. By inpainting stroke lesions, SynthSR enables accurate segmentation with existing packages like FreeSurfer (Fig. 6A). Comparing the ipsi- and contralateral volumes of five representative ROIs (hippocampus, amygdala, thalamus, putamen, and caudate; see Table 3) reveals asymmetry patterns that are consistent with the literature, e.g., no asymmetry in the hippocampus (*71*), but strong asymmetry in the thalamus (*72*) and the other ROIs. Table 3 also shows the correlation between the volume of the stroke lesion and that of the ROIs in the ipsi- and contralateral sides. The hippocampus shows no statistically significant correlation on either side, while the other ROIs display weak negative correlation on the ipsilateral side but no significant correlation on the contralateral side. The strength of these correlations (*r* ~ −0.4) is consistent with values reported in other studies, e.g., *r* = −0.3 in (*73*).

**Table 3.** Volume of subcortical ROIs (ipsilateral and contralateral) is stroke. This table shows the mean volume of several representative ROIs in the contralateral and ipsilateral side of the stroke, the effect size between the two distributions (i.e., the asymmetry), and the correlation between the stroke lesion size and the volumes of the ROIs, including *P* values computed with Student’s *t* test. We only included in this analysis stroke lesions that had more than two-thirds of their volume in one of the two hemispheres (*N* = 598).

Region	Hippocampus	Amygdala	Thalamus	Putamen	Caudate
Contralateral volume, mean and SD (mm^3^)	4861 ± 560	2113 ± 274	7131 ± 803	5442 ± 686	4551 ± 483
Ipsilateral volume, mean and SD (mm^3^)	4831 ± 560	1999 ± 310	5958 ± 1420	4642 ± 1147	4096 ± 655
Effect size: Contralateral versus ipsilateral	0.05	0.39	1.02	0.85	0.79
Correlation of lesion and contralateral volumes and *P* value	0.011 (*P* = 0.78)	−0.027 (*P* = 0.50)	0.021 (*P* = 0.61)	0.052 (*P* = 0.20)	0.07 (*P* = 0.07)
Correlation of lesion and ipsilateral volumes and *P* value	0.003 (*P* = 0.93)	−0.300 (*P* < 10^−13^)	−0.410 (*P* < 10^−24^)	−0.494 (*P* < 10^−37^)	−0.349 (*P* < 10^−17^)

SynthSR can also improve registration of these images, which finds application in, e.g., construction of templates for spatial normalization and voxel-wise analysis, for longitudinal analysis, or to correct for edema (*74*). We performed the atlas-building experiment from the previous section and obtained the templates in Fig. 6B. The average lesion is centered in the basal ganglia and deep white matter. One possible explanation would be an overrepresentation of small vessel strokes in the ATLAS dataset, since these are prevalent in the thalamo-capsular region. The lesion map also shows that strokes are on average larger on the right hemisphere, since the heatmap is stronger and the total number of left and right hemispheric strokes is approximately the same. Once again, further assessment will be required to determine whether this is an actual effect or the consequence of a selection bias, e.g., large lesions on the left hemisphere may affect speech and lead to reduced enrollment rates in ATLAS.

## DISCUSSION

We present a publicly available AI technique (SynthSR) that can transform any clinical brain MRI scan into a synthetic 1-mm MPRAGE that is compatible with practically every image analysis method for 3D morphometry of human brain MRI. Because SynthSR works out of the box, combining it with existing tools is a cost- and time-efficient alternative to designing algorithms for a specific type of MRI acquisition (orientation, resolution, and contrast). Such an approach not only would require human resources and specific anatomical and ML expertise to compile labeled datasets and train CNNs but also would be impractical when analyzing heterogeneous datasets. We note that, while SynthSR is compatible with any MRI acquisition, the quality of the output is higher for inputs with HR and contrast. SynthSR is publicly available as part of our widespread open-source neuroimaging software FreeSurfer, has no tunable parameters, and runs out of the box in a few seconds, making it very easy for anybody to use.

We have shown that SynthSR can be used in combination with the segmentation modules in FreeSurfer or FSL to chart trajectories of ROI volumes in aging and distinguish AD from control brains using a highly heterogeneous dataset of clinical scans, and we replicated the results of prior studies that used large numbers of research scans. This was made possible by the strong generalization ability provided by the DR strategy at training. Combined with a standard registration package like NiftyReg, SynthSR can also accurately register clinical data, including challenging cases, like scans of patients with glioma or stroke.

By facilitating analysis of brain MRI with existing tools, SynthSR has both clinical and research applications. In the clinic, SynthSR has the potential to enable 3D morphometry of anisotropic acquisitions (e.g., to measure longitudinal change, as in the BraTS-Reg experiment). In research, it has the potential to examine a variety of questions and may greatly increase the statistical power of many types of studies. For example, in studies of early disease stages, the increased sample sizes may enable the detection of subtle effects. Another example is the application to genome-wide and brain-wide association studies, where the required sample sizes are well into the thousands (*21*). SynthSR may also be used to facilitate the inclusion of minorities, which are often underrepresented in prospective studies but are more frequently scanned for clinical purposes.

SynthSR promises to increase the reproducibility of neuroimaging studies in two different manners: first, by being publicly available and not requiring any training or fine tuning; second, because it will deliver an increase in sample sizes that will, in turn, increase the reproducibility of studies. We emphasize that the purpose of SynthSR is not to produce images for clinical diagnosis but rather to generate synthetic scans with inpainted abnormalities that any neuroimaging software for 3D morphometry can use.

SynthSR has two main limitations. The first one is the fact that every tissue type (including pathological tissue) requires a label in the model. This is problematic when modeling continuous processes, e.g., tumor infiltration, which creates continuous gradients in the image intensities (due to partial voluming) that SynthSR does not model. Examples of this phenomenon are shown in fig. S2 (A to D). In future work, we plan to address this issue by compiling a dataset of lesions segmented at a finer level of detail and using it to retrain SynthSR, and also by using more complex models of pathology. The second limitation is that SynthSR is not guaranteed to yield plausible images, which can be problematic when inpainting large lesions. This is illustrated in fig. S2 (E and F), where SynthSR not only successfully inpaints a large stroke with white matter and cortical tissue but also hallucinates a smooth white matter boundary as well as gray matter in the contiguous inferior lateral ventricle. These problems could potentially be circumvented with additional GAN losses or by trying to quantify uncertainty.

In addition to tackling these two problems, future work will also explore more complex deep learning architectures. While we used a U-net (*75*, *76*) that provides state-of-the-art results in many medical image segmentation and regression problems, the quick progress in architectures driven by the ML community will likely enable us to improve SynthSR with more sophisticated architectures in the coming years (e.g., transformers). Because millions of clinical brain MRI scans are acquired worldwide every year, we believe that tools that make them usable for quantitative analysis like SynthSR have the potential to transform human neuroimaging research and clinical neuroradiology.

## MATERIALS AND METHODS

SynthSR relies on a CNN that requires a dataset of 1-mm isotropic 3D MPRAGE scans and companion neuroanatomical labels for training. Then, the trained CNN can be used to analyze any other dataset. In this section, we first describe the datasets used in training and in the experiments leading to the results reported above. Then, we describe the methods to train SynthSR and apply it to new data.

### Datasets

#### Training dataset

Our approach, SynthSR, is trained on a subset of twenty 1-mm isotropic 3D MPRAGE scans from the Open Access Series of Imaging Studies (OASIS) dataset (*77*), with dense neuroanatomical labels for cerebral and extracerebral ROIs. The cerebral ROIs comprise 36 brain regions and were manually labeled by members of our laboratory in previous work (*8*). The extracerebral regions were automatically obtained with a Bayesian approach using the atlas presented in (*78*), which includes labels for the eyes, skull, and soft tissue. The 20 subjects include 14 healthy volunteers and 6 subjects with probable AD, aged 51.3 ± 27.5 years (range, 18 to 82).

#### Test datasets

The experiments above rely on five different datasets. The first dataset comprises 9146 brain MRI scans from 1110 sessions, downloaded from the PACS at MGH under Institutional Review Board approval. The sessions correspond to subjects who had memory complaints and are not expected to have big lesions due to, e.g., tumors or strokes. This dataset is uncurated and includes a wide array of MRI modalities, including structural, angiography, and diffusion, among others. Virtually, every session is different from all others and includes different numbers of scans with different MRI modalities, contrasts, resolution, orientation, slice spacing, and thickness. Scans with more than three dimensions (typically diffusion) or with ICV under 1.1 liters (typically with partial field of view) were left out of the dataset. We refer to this dataset as the MGH dataset. In addition, we used four large public datasets that cover three different types of pathology: ADNI (*5*), ATLAS (*69*), and BraTS/BraTS-Reg (*60*, *63*), which include subjects with AD, stroke, and brain tumors, respectively. Further details on the acquisitions can be found in the corresponding publications.

### Data generation

#### Related work

SynthSR builds on our prior work on joint SR and synthesis using a combination of real and synthetic MRI scans (*44*). For the sake of completeness, we summarize our previous approach here. The method used a dataset of real 3D MPRAGE scans (with normalized, bias field–corrected intensities) and companion dense segmentations (including extracerebral tissue) to train a regression 3D CNN—a U-net (*75*, *76*)—as follows. At every iteration, a random scan and corresponding segmentation were selected and geometrically augmented with a combination of random linear and nonlinear deformations (the latter, diffeomorphic). The deformed segmentation was then used to generate a synthetic scan with a generative model inspired by that of Bayesian segmentation, i.e., a Gaussian mixture model (GMM) conditioned on the underlying labels, combined with a model of additive noise and multiplicative bias field (*10*). Specific orientation, slice spacing, and slice thickness were then simulated using a model of partial voluming and subsampling (*79*). The CNN weights were then updated using this LR synthetic scan as input, and the 1-mm isotropic MPRAGE scan as ground-truth output. The GMM and resolution parameters were set to mimic the appearance of an arbitrarily prescribed type of acquisition, possibly multimodal (e.g., 4-mm coronal T2 plus 5-mm axial FLAIR).

**Fig. 7.**
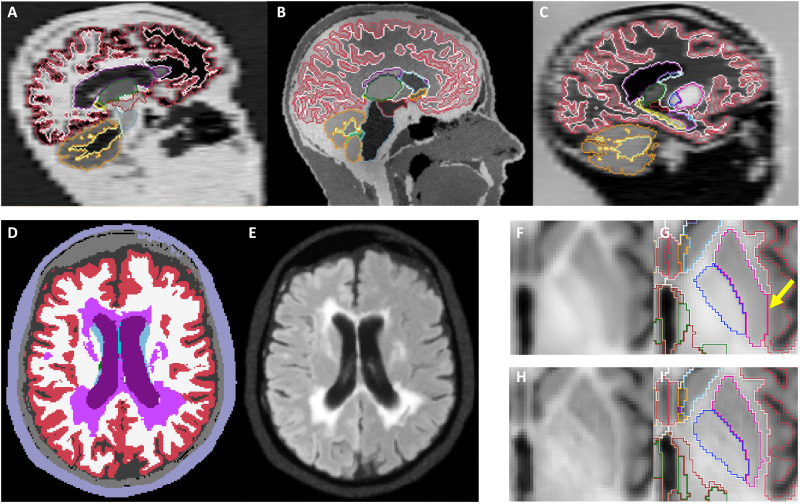
Key improvements in SynthSR. Domain randomization: Sagittal slices of three synthetic samples with different orientation, contrast, and resolution are shown, along with the segmentations that were used to simulate them (**A** to **C**). Lesion modeling: Label maps augmented with lesion labels (**D**) are used to generate images with pathology (**E**). Auxiliary segmentation CNN: Blurry features are often obtained when minimizing only the error in estimated intensities (**F**), leading to segmentation errors (**G**). Encouraging the output image to be more accurately segmentable by a previously trained CNN noticeably sharpens the output (**H**) and ultimately leads to better segmentation (**I**).

As mentioned in Introduction, our newly proposed method addresses the three limitations of our previous approach, because of three key improvements (Fig. 7)—two of them related to data generation (discussed here), and a third related to the CNN architecture (in the next subsection).

#### Domain randomization

In our previous work, the synthetic scans used as input during training sought to mimic a specific type of acquisition. While this strategy enabled training of CNNs without specific training data (e.g., one could easily change the simulated spacing to 4 mm), it produced CNNs that were tailored to a specific acquisition. Here, we instead adopt a DR approach. DR has recently shown great success in robotics and computer vision (*41*). DR relies on training on a huge variety of simulations with fully randomized parameters—in our case, MR contrast (means and variances of ROIs), orientation (sagittal, axial, and coronal), slice spacing, slice thickness, bias field, and noise level (Fig. 7, A to C). By exposing the CNN to a different contrast, orientation, resolution, and noise level at every iteration during training, it learns not to rely on such features when estimating the output, thus becoming agnostic to them. Moreover, DR effectively addresses catastrophic forgetting (i.e., the fact that learning a new task may degrades performance in older task) (*80*) such that continual learning methods (*81*) are not required. The practical implications of DR on SynthSR are huge, since it bypasses the need to retrain and enables processing of heterogeneous datasets with a single CNN—all with a single FreeSurfer command:

mri_synthsr --i [input_scan] --o [synthetic_1mm_isotropic_t1]

#### Robustness to pathology

Modeling of pathology has two main aspects: shape and appearance—both of lesions and normal tissue. SynthSR seeks to preserve changes in shape of ROIs due to disease (e.g., atrophy) such that they can be captured by subsequent analyses to study clinical populations, e.g., as in the AD volumetry experiment above. This is achieved with a combination of two strategies: using a diverse training dataset including subjects with a wide age range (18 to 82 years) and pathological atrophy (Alzheimer’s), and aggressive shape augmentation of the 3D segmentations during training with random diffeomorphic deformations.

In terms of appearance, we have trained SynthSR to inpaint pathology with normal tissue to enable subsequent analysis with any morphometry tool. This is crucial when using methods that falter in the presence of lesions. Examples include the segmentation or registration of scans with strokes or tumors (as in our experiments with BraTS-Reg and ATLAS) or neuroimaging studies of multiple sclerosis, where it is common to segment white matter lesions and fill them with synthetic intensities resembling healthy white matter before processing with FreeSurfer or SPM (*82*, *83*). To achieve this effect, we train the CNN with inputs where we randomly synthesize pathology, and outputs where no pathology is present. This is achieved as follows. First, we enhance the label maps of the training dataset with lesion segmentations registered from 60 held-out cases from ADNI, BRATS, and ATLAS (Fig. 7D). These new lesion labels are just treated as additional ROIs during image generation, creating synthetic lesions of random appearance during training (Fig. 7E). Second, we ensure that the regression targets (i.e., the MPRAGE images of the training dataset) do not display any pathology. Because these scans show some white matter lesions, we segment them with an open-source Bayesian method (*84*) and inpaint them with a publicly available algorithm (*83*) to obtain “clean” scans.

#### Generalization ability

The proposed method is extremely robust to overfitting. In terms of image intensities, SynthSR is practically immune to this phenomenon because it is trained with synthetic images generated with a model whose parameters (means, variances, and bias field) are fully randomized at every iteration during training. In terms of shape, overfitting is prevented by the aggressive geometric augmentation scheme described above, which includes strong random nonlinear deformation of the 3D segmentations and random registration of random lesions at every iteration. Therefore, images of any orientation, resolution, and contrast can be fed to SynthSR without any preprocessing (e.g., no skull stripping or bias field correction is required), other than a simple minimum-maximum normalization of the intensities to comply with the range of inputs expected by the CNN.

### CNN architecture

Our CNN builds on the architecture of our previous work, which is essentially a regression 3D U-net (*75*, *76*). The U-net architecture is widespread in medical image segmentation and produces state-of-the-art accuracy in a wide array of tasks (*85*). Our previous work used a single CNN to regress the MPRAGE intensities from the input. However, this strategy often led to errors that, albeit small in terms of loss, could lead to large mistakes in subsequent tasks. For example, small errors in the predicted intensities may not influence the segmentation in the middle of the large structures, but can easily lead to mistakes around structures with faint boundaries (Fig. 7, F and G).

Here, we add a second CNN to the architecture: a segmentation U-net that is concatenated to the output of the regression U-net. This segmentation U-net is trained in advance, in a supervised fashion, using the training dataset (i.e., it predicts segmentations from MPRAGE scans). This CNN is frozen during training of SynthSR such that the regression network is encouraged to synthetize images that can be accurately segmented by the supervised network, thus solving many of the problems of training only with regression (Fig. 7, H and I).

The regression and segmentation CNN have the same architecture, except for the final layer: The regression CNN has a linear layer with one feature, whereas the segmentation CNN has as many features as labels to segment, which are turned into probabilities with a softmax function. Both networks are 3D U-nets with five resolution levels, each consisting of two layers; each layer comprises a convolution with a 3 × 3 × 3 kernel and a nonlinear exponential linear unit (ELU) activation. The first layer has 24 features. The number of features is doubled after each max pooling and halved after each upsampling. The two U-nets are concatenated with the synthetic data generator into a single model completely implemented on the GPU for fast training, using Keras/TensorFlow.

### Learning and inference

Training of our network minimizes a linear combination of two losses: a regression loss and a segmentation loss, combined with a relative weight λ. The goal is to minimize this loss with respect to the neural network parameters θ

$$\hat{\theta }=\underset{\theta }{argmin}L(\theta ;X,Y,L_{y})=L_{\mathrm{reg}}(\theta ;X,Y)+\lambda \;L_{\mathrm{seg}}(\theta ;X,L_{y})$$

where $\hat{\theta }$ represents the optimal parameters, *X* is the input image, *Y* is the normalized ground-truth MPRAGE (with mean white matter intensity matched to 1.0), and *L_y_* is the ground-truth segmentation of the MPRAGE. The regression loss is given by the L1 norm, i.e., the sum of absolute differences of the ground truth and predicted intensities

$$L_{\mathrm{reg}}(\theta ;X,Y)={\left\|Y-f(X;\theta )\right\|}_{1}$$

where *f*(*X*; θ) is the prediction for input image *X* when the CNN weights are equal to θ. The L1 norm produces crisper results than its L2 counterpart, i.e., sum of squared differences (*44*).

The segmentation loss is given by the (negated) soft average Dice score (overlap) between the ground-truth segmentation and the labels predicted by the segmentation U-net when given the predicted intensities (*86*)

$$L_{\mathrm{seg}}=1-\mathrm{DICE}[L_{y},\mathrm{SEG}(f(X;\theta ))]$$

where SEG( · ) represents processing with the segmentation network. We emphasize that the use of these direct losses is much less likely to “hallucinate” features than those based on GANs, which produce highly realistic details that may not always be real (*87*).

This training loss is minimized using the Adam optimizer (*88*) with a learning rate of 10^−4^ and 250,000 iterations (approximately 2 weeks on an RTX 8000 GPU), after which the loss has converged by all practical measures. We set the relative weight to λ = 0.25 by visual inspection of the results on a small held-out dataset. The synthetic images are minimum-maximum normalized to the [0,1] interval and up-scaled to 1-mm isotropic resolution such that the input and output images live in the same voxel space.

After training, one simply strips the synthetic generator and segmentation CNN from the model, as they are not needed for inference at test time. Input volumes are up-scaled to a resolution of 1 mm × 1 mm × 1 mm, padded to the closest multiple of 32 voxels in each of the three spatial dimensions (required by the five resolution levels), minimum-maximum normalized to [0, 1], and processed with the regression CNN to estimate the synthetic MPRAGE intensities at 1-mm isotropic resolution. Inference takes less than 3 s on an RTX 8000 GPU and approximately 20 s on a modern CPU.

### Statistical methods

We used Shapiro-Wilk tests (*53*) to assess whether data samples followed Gaussian distributions. Because this was never the case in our experiments, we used nonparametric statistics to compare populations: Wilcoxon rank sum tests to compare the medians (*54*) instead of *t* tests, and AUROCs instead of effect sizes.
