# Supplementary material for: SynthSR: A public AI tool to turn heterogeneous clinical brain scans into high-resolution T1-weighted images for 3D morphometry
Source: Sci Adv. 2023 Feb 1;9(5):eadd3607. doi: 10.1126/sciadv.add3607 (PMC9891693; doi:10.1126/sciadv.add3607)
Supplement: Supplementary file 1 — Figs. S1 and S2 [file sciadv.add3607_sm.pdf]

Science Advances
AAAS

Supplementary Materials for

SynthSR: A public AI tool to turn heterogeneous clinical brain scans into high-resolution T1-weighted images for 3D morphometry

Juan E. Iglesias *et al.*

Corresponding author: Juan E. Iglesias, jiglesiasgonzalez@mgh.harvard.edu

Sci. Adv. **9**, eadd3607 (2023)
DOI: 10.1126/sciadv.add3607

The PDF file includes:

Figs. S1 and S2
Legends for data file S1

Other Supplementary Material for this manuscript includes the following:

Data file S1

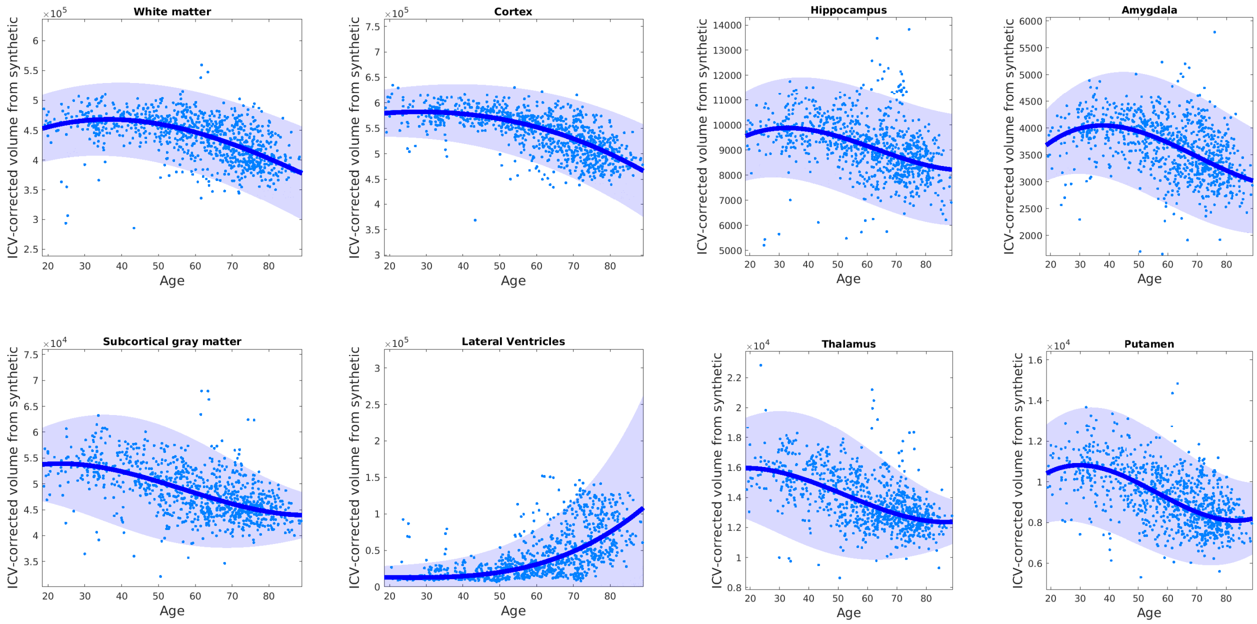

Fig. S1. Brain volume trajectories derived from clinical data using *SynthSR* combined with FreeSurfer (at subject level). Clinical scans were processed with *SynthSR* and then segmented with FreeSurfer to obtain ROI volumes, as well as an estimate of the intracranial volume (ICV). The ROI volumes were corrected by ICV and gender, and regressed against age using a Laplace distribution with location and scale modeled with a spline with six knots. The Laplace distribution provides a more robust fit than a Gaussian. The median trajectory is overlaid on the individual (ICV- and gender-corrected) volumes; the 95% confidence interval is shaded. Every point represents a volume for one subject, which is computed as the median of the volumes derived from all available scans for that subject. Trajectories at the individual scan level are shown in the main manuscript (Fig. 2).

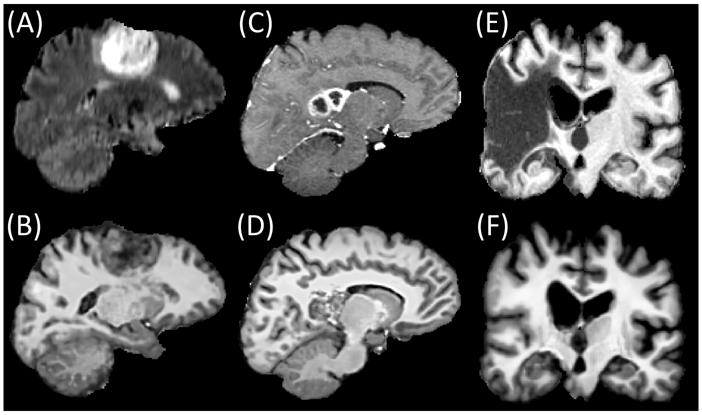

Fig. S2. Failure cases in presence of pathology. (A) Sagittal slice of FLAIR scan from BraTS-Reg with tumor in motor cortex. (B) Output of *SynthSR*; the algorithm mistakes the inferior, anterior, and posterior boundaries of the tumor by white matter, and the center of the tumor by cerebrospinal fluid. (C) Sagittal slice of a contrast-enhanced T1 scan from BraTS-Reg. (D) *SynthSR* replaces the tumor with cortex-like features, rather than cerebrospinal fluie and white matter. (E) Coronal slice of a T1 scan from ATLAS. (F) *SynthSR* replaces the large stroke lesion with plausible tissue, albeit with excessively smooth boundaries between white and gray matter. Moreover, it hallucinates gray matter in the inferior lateral ventricle contiguous to the lesion (above the hippocampus).

Data File S1: Spreadsheet with list of landmarks that passed QC in Brats-Reg experiment.

The first column is the name of the case, as specified in the data release of BraTSReg. The second column is the landmark number, following the same order as in the original data release. The third column is the result of the QC: pass (1) or fail (0).
